# Interferon targeted therapies in systemic lupus erythematosus

**DOI:** 10.31138/mjr.28.1.13

**Published:** 2017-03-28

**Authors:** Durga Prasanna Misra, Vir Singh Negi

**Affiliations:** 1 Department of Clinical Immunology, Sanjay Gandhi Postgraduate Institute of Medical Sciences (SGPGIMS), Lucknow, India,; 2 Department of Clinical Immunology, Jawaharlal Institute of Postgraduate Medical Education & Research (JIPMER), Puducherry, India

**Keywords:** Type I interferon, systemic lupus erythematosus, sifalimumab, rontalizumab, interferon alpha kinoid, plasmacytoid dendritic cell

## Abstract

Type I interferons secreted by plasmacytoid dendritic cells (pDCs) play a crucial role in the pathogenesis of systemic lupus erythematosus by driving the formation of autoantibodies against nuclear debris. Inherited mutations causing activation of the Type I interferon pathway result in a phenotype of systemic autoimmunity which resembles some of the manifestations of lupus. Patients with lupus have increased expression of interferon-stimulated genes in the peripheral blood mononuclear cells which is abrogated following immunosuppressive treatment. Recent therapeutic approaches have involved monoclonal antibodies directly targeting interferon alpha (sifalimumab, rontalizumab) or the use of interferon alpha kinoid to stimulate endogenous production of anti-interferon antibodies in lupus. Other drugs used in lupus such as hydroxychloroquine and bortezomib also reduce circulating levels of type I interferons. Newer therapeutic strategies being investigated in preclinical models of lupus that reduce the production of Type I interferons include dihydroartemisinin, Bruton’s tyrosine kinase antagonists, Bcl-2 antagonists and sphingosine-1 phosphate agonists.

Interferons, originally identified as inhibitors of viral replication, are cytokines produced by immune cells. There are three families of interferons viz. Type I, Type II and Type III interferons. Type I interferons, which are predominantly involved in immune responses against viral infections, include interferon alpha (IFN-α), interferon beta (IFN-β), IFN-kappa, IFN-epsilon and IFN-omega. Type II interferon (IFN-gamma) is predominantly involved in immune responses against intracellular bacteria such as *Mycobacterium tuberculosis*. Type III interferons include interferon lambda (λ1, λ2 and λ3).^1^

Evidences in literature suggest that viral infections such as Epstein-Barr virus (EBV) may have a role in driving pathogenesis of systemic lupus erythematosus (SLE), which is a multisystem autoimmune disease associated with significant morbidity and higher risk of mortality, most commonly affecting young females. Patients with childhood-onset SLE have higher titres of antibodies to EBV compared to healthy controls.^2^ Cross-reactivity between antibodies to EBV nuclear antigen 1 (EBNA1) and anti-Ro 60 antibodies (which are often found early in the course of lupus) suggests a role for EBV in driving the initiation of lupus.^3^ One of the mechanisms linking viral infections and lupus is that both of these are associated with higher circulating levels of Type I interferons. Indeed, Type I interferon plays a critical role in the pathogenesis of pristane-induced lupus, which is a robust animal model of this disease. Disease phenotype is ameliorated in this lupus model by knocking out receptors for Type I interferon.^4^ Genes involved in regulation of interferon signaling such as *IRF 5*, *IRF 7*, *IFIH 1* and *STAT 4* have been identified as risk factors for SLE across different populations.^5^ A group of inherited diseases characterized by high circulating levels of Type I interferons (called the Type I interferonopathies) have been recently identified. These involve defects in genes encoding for proteins involved in nucleic acid (deoxyribonucleic acid – DNA, ribonucleic acid - RNA) clearance such as *TREX 1*, *RNASEH 2A* and *RNASEH 2B*. These result in a multisystem autoimmune disease (Aicardi Goutierre syndrome) with cutaneous and neurologic involvement.^6^ Mutations in *TREX 1* also predispose to lupus.^6^ In humans, Type I IFN is mostly produced by plasmacytoid dendritic cells (pDCs).

Apoptotic defects have been identified as a key mechanism in the generation of autoantibodies in lupus. Normally, apoptotic debris is cleared before it could attract the attention of the immune system. In lupus, apoptotic debris containing nuclear material (which is normally not exposed to the immune cells) persists due to defects in clearance due to complement deficiencies, defective enzymes responsible for cleaving nucleic acids (such as DNAse and RNAse) and defects in Fcγ receptors. These are now taken up by antigen presenting cells, which in turn present them to B and T lymphocytes, ultimately resulting in formation of plasma cells that produce antibodies to these nuclear antigens (antinuclear antibodies, which characterize lupus). These autoantibodies further bind to existing apoptotic debris, forming antigen-antibody complexes which now act on Toll-like receptors on the surface and well as intracellularly inside endosomes of the pDCs (DNA acts on TLR9 and RNA acts on TLR3, TLR7, TLR8). In turn, via IRF5, IRF7 and other downstream mediators, this results in stimulation of interferon-stimulated genes (ISGs) which result in increased secretion of Type I IFN. Type I IFN further acts to promote autoimmunity mediated by B and T lymphocytes, including production of autoantibodies^7,8^ (**Figure 1**). The critical nature of this mechanism in the pathogenesis of lupus is emphasized by the fact that scavenging nucleic acid debris has emerged as the newest therapeutic modality being investigated in animal models of lupus.^9^

**Figure 1:**
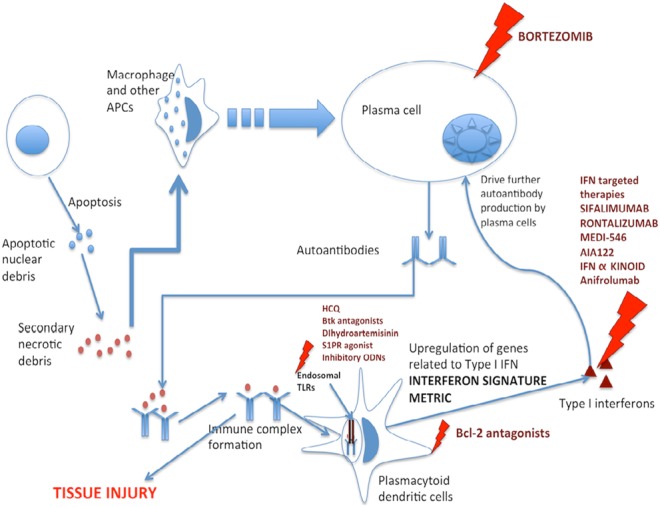
Genesis of Type I interferons and their targeting APC – Antigen presenting cells, HCQ – Hydroxychloroquine, IFN – Interferon, ODN – oligonucleotide, S1PR- Sphingosine-1-phosphate receptor, TLR – Toll-like receptor.

Early depletion of pDCs in another murine model of lupus resulted in significant amelioration of the phenotype of lupus which was associated with reduced transcription of genes induced by IFN-α and IFN-β^10^, highlighting the importance of pDCs and Type I IFN in the pathogenesis of lupus. Systemic lupus erythematosus is associated with increased risk of incident cardiovascular events.^11^ Type I IFNs have been shown to promote endothelial dysfunction, plaque instability and promote plaque rupture in murine models of lupus,^12^ hence may be contributory towards the observed increase in cardiovascular events in patients with lupus.

Increased expression of genes expressed downstream to interferon signaling has been detected in lupus as well as in other autoimmune diseases such as polymyositis, dermatomyositis, rheumatoid arthritis and systemic sclerosis.^13^ The United States Food and Drug Administration (USFDA) is the major regulatory authority for drugs in the United States of America and is considered a reference for therapeutics worldwide. Very few therapies are approved by the USFDA for the treatment of lupus including hydroxychloroquine and belimumab (a monoclonal antibody targeting the B-lymphocyte stimulator).^14^ Hydroxychloroquine acts to decrease interferon signature in the peripheral blood lymphocytes of patients with lupus.^15^ Of late, direct targeting of Type I IFN in lupus has been the focus of numerous pre-clinical and clinical studies in lupus over the past few years. Considering the spate of recent trials on molecules targeting this pathway, we decided to review the recent literature (over the past five years) on interferon-targeted therapies in SLE.

## SEARCH STRATEGY

The search strategy advocated by Gasparyan et al. was adhered to.^16^ The database MEDLINE/PubMed was searched using terms “Type I interferons” and “lupus” on the 25^th^ of October 2016 for articles published over the past five years. 361 items were retrieved and screened to identify those describing the targeting of Type I interferons in experimental models or human subjects with lupus. Using the same search terms, an additional search on Scopus on the 15^th^ of November 2016 retrieved 503 articles, and a search on Cochrane Central Register of Controlled Trials retrieved 18 results. These new searches identified no new published clinical trials from those already identified on the search on MEDLINE/PubMed. Published original articles were included; case reports and review articles were excluded. We did not include conference abstracts that had not been already published as original articles.

## INTERFERON SIGNATURE METRIC

In order to understand the targeting of Type I IFN in lupus, it is important to understand the interferon signature metric. It was demonstrated that patients with active lupus have a higher expression of genes whose expression is induced by Type I IFN. Bennett et al.^17^ analyzed 30 patients with pediatric lupus (pSLE) and compared them with nine healthy controls and 12 patients with juvenile idiopathic arthritis (JIA). Peripheral blood mononuclear cells (PBMC) from these subjects were isolated and ribonucleic acid (RNA) from these cells was extracted and subjected to microarray analysis to identify gene expression. They identified 26 such Type I IFN-induced genes in patients with pSLE compared with healthy controls and those with JIA. Eight of these Type I IFN-induced genes correlated with disease activity in patients with pSLE assessed by the SLE disease activity index (SLEDAI). Treatment with high dose intravenous methylprednisolone pulses for three days in three patients with active lupus extinguished this Type I IFN signature. Baechler et al^18^ further identified genes upregulated by interferons by treating PBMC from healthy individuals with interferons in vitro and assessing the induced genetic signature by microarray analysis of extracted RNA from these cells. The expression of these interferon-stimulated genes when quantified electronically constituted the interferon signature, which they further studied in 48 patients with lupus compared with 42 healthy controls. The interferon signature was significantly higher overall in patients with lupus compared with healthy controls. Approximately three-fourths of their patients with lupus had this interferon signature, and these patients had higher proportion of renal, neurological or hematological involvement compared with those without.

Quantitatively analyzing a large number of genes is cumbersome and costly. It was possible that a smaller number of genes may accurately reflect the overall burden of genes induced by Type I interferons. Hence, Kennedy et al.^19,20^ identified three genes (*EPSTI1*, *HERC5* and *TYK1*) amongst 128 genes upregulated in response to Type I interferons. The quantitative expression of these three genes (i.e., the Interferon signature metric or ISM) strongly correlated with the quantitative expression of the 128 aforementioned genes taken together (Spearman’s rho > 0.96). Hence, the ISM generated using these three genes was a surrogate for gene expression induced by Type I interferons. Furthermore, on multivariate analysis, they identified that shorter disease duration, positivity for anti-double stranded DNA antibodies and antibodies to extractable nuclear antigens, serum levels of B cell activating factor (BAFF) and lower CD4+ lymphocyte counts associated with the ISM. ISM may be derived using as many number of genes as is feasible in a particular study and not just these three genes. This ISM has been used to identify those patients with high interferon-inducible gene expression who may be candidates for therapy with drugs directly targeting Type I IFNs, as we shall subsequently discuss. It is important to note that the different types of interferon signature metric described in different studies vary from each other. Hence, before embarking on a study intending to utilize the ISM, one must run a microarray of interferon induced genes in the population of interest and attempt to derive an ISM based on as many genes as are feasible to be studied repeatedly depending on the available resources, for that particular population and that particular study. **Table 1** summarizes the different genes used in derivation of the ISM in the different studies assessing the utility of interferon-targeted therapies in lupus.

**Table 1:** Genes implicated in the derivation of interferon signature in different studies on interferon targeted therapies in lupus

Reference No.	Drug	Genes involved in interferon signature
23	Sifalimumab	*IFI27, IFI44, IFI44L, RSAD2*
24, 25	Rontalizumab	*IFI27, IFI44, MX1, IFIT1, OAS1, OAS2, and OAS3*
30	Interferon α kinoid	Score derived using 21 genes including *IFI27, IFI44L, EPSTI1* and *RSAD2*

## AGENTS DIRECTLY TARGETING TYPE I INTERFERONS

We could identify two phase I studies and one phase II study on sifalimumab, one phase I and one phase II study on rontalizumab and a single phase I/II study on interferon-alpha kinoid in lupus.

Sifalimumab is a fully human monoclonal IgG1κ antibody against interferon alpha. In a phase I study,^21^ 50 patients with SLE receiving a single dose of intravenous sifalimumab (0.3, 1, 3, 10 or 30 mg/kg) were compared with 17 patients treated with placebo and found to have similar adverse effect profile, including no increased risk of infections. Those receiving sifalimumab had improvement of SLEDAI scores when compared with worsening of SLEDAI scores in those receiving placebo. Those patients with a high interferon signature metric had reduction of the same following treatment with sifalimumab; thus, the reduction of IFN-α/β induced proteins in the skin biopsy of a patient with SLE was also observed after administration of the drug. In another phase I study,^22^ 121 patients with active lupus were treated with intravenous sifalimumab at varying doses between 0.3, 1, 3 and 10 mg/kg 2 weekly for 14 doses, with 40 patients receiving placebo. No increase in adverse effects was observed in those patients receiving sifalimumab. When adjusted for use of steroids to treat flares, patients receiving sifalimumab had a significant improvement in mean scores of Safety of Estrogens in Lupus Erythematosus National Assessment SLEDAI (SELENA-SLEDAI) compared to those receiving placebo. Sifalimumab was more effective than placebo in normalizing complement levels. A phase IIb trial of sifalimumab in SLE was recently reported.^23^ In this study, 324 patients with active lupus were treated with intravenous sifalimumab at doses of 200 mg, 600 mg or 1200 mg (day 1, day 15, day 29 followed by every 28 days) compared with 107 patients receiving placebo. Higher proportion of patients receiving sifalimumab at all doses individually attained the primary end-point of the SLE Responder Index (SRI) when compared with placebo. Those with significant cutaneous involvement had better improvement in the cutaneous lupus erythematosus area and severity index (CLASI) compared to placebo; better improvement of fatigue scores was also noted. Patients receiving sifalimumab had lesser disease flares, better physician global assessment scores, greater improvement in SLEDAI 2000 (SLEDAI-2K) scores and response when assessed using the British Isles Lupus Assessment Group (BILAG)-based Composite Lupus Assessment (BICLA) criteria compared to placebo. In this study, ISM was assessed using four genes. Those with a high baseline ISM had significant improvement in lupus disease activity scores compared to placebo. However, the small number of patients with low ISM meant that comparisons between ISM^high^ and ISM^low^ subgroups were not feasible. The adverse effect profile was similar in patients in both the groups.

Rontalizumab is another humanized IgG1 monoclonal antibody which neutralizes all subtypes of IFN-α. In a phase I dose ranging study,^24^ rontalizumab was studied in 48 patients with SLE compared with 12 others receiving placebo. The doses used with gradual escalation under careful monitoring were 0.3mg/kg intravenously (iv), 1 mg/kg iv and subcutaneous (sc), 3 mg/kg iv and sc and 10 mg/kg iv. There were no significant differences in the incidence of mild, moderate and serious adverse events (including infections) in between patients receiving rontalizumab or placebo. Notably, one of the patients receiving rontalizumab developed acute myeloid leukemia. Regarding interferon signature, the ISM for this particular study was calculated using expression of seven IFN-inducible genes. A dose-dependent reduction in the ISM score as observed after administration of the first as well as subsequent doses of rontalizumab, with reduction in circulating levels of proteins known to be induced by interferons such as monocyte chemoattractant protein 1 (MCP1). No significant reduction in circulating autoantibodies was noted following treatment with rontalizumab. Based on the promise shown by the phase I study, a phase II clinical trial of rontalizumab in SLE (ROSE trial) was conducted.^25^ In this study, 159 patients with lupus were randomized to receive rontalizumab (81 received 750 mg iv monthly for 20 weeks, 78 received 300 mg sc 2 weekly for 22 weeks) and compared with 79 placebo-treated patients. All patients on placebo, 79 on iv rontalizumab and 77 on sc rontalizumab completed follow-up till 24 weeks. The study did not meet the pre-specified end point, i.e., similar proportion of patients receiving rontalizumab and placebo attained response as defined by the BILAG responder index or the secondary end point of SRI, irrespective of whether they were ISM^high^ or ISM^low^. Paradoxically, secondary analysis revealed that those patients having a low ISM had decreased number of flares on rontalizumab, and more of them were able to attain doses of prednisolone ≤ 10 mg/day compared to placebo. However, this was not seen in those with a high ISM. No significant difference was observed in adverse effects in between the groups receiving rontalizumab and placebo. The SRI response rates with sifalimumab and rontalizumab are comparable with those achieved with belimumab.^26,27^

Another novel approach is to stimulate endogenous production of neutralizing antibodies to interferon α. This is accomplished by the interferon α kinoid (IFN-K), wherein patients with lupus are immunized with IFN-α conjugated with keyhole limpet hemocyanin to enhance its antigenicity. The ability of this approach to generate neutralizing antibodies that ameliorated the phenotype of lupus was initially shown in NZB/W F1 mice, which serve as a murine model of lupus.^28,29^ This approach was replicated in humans in a phase I/II dose escalation placebo-controlled study of 28 patients with lupus.^30^ Such antibodies to IFN-α generated endogenously by immunizing with IFN-K administered intramuscularly at doses of 30 μg, 60 μg, 120 μg or 240 μg administered at day 0, day 7, day 28 and day 84 (the last dose was only given in the groups receiving IFN-K at a dose of 60 μg or greater, if neutralizing antibodies to IFN-α had not been detectable in serum at day 56. At all doses, treatment with IFN-K resulted in circulating antibodies to IFN-α when compared with placebo. However, neutralizing antibodies to IFN-α were not detected in those receiving IFN-K at a dose of 30 μg. Threee out of 6 patients each receiving the 60 μg and 120 μg doses of IFN-K, and 4 out of 5 patients treated with 240 μg IFN-K developed neutralizing antibodies to IFN-α. This study used a ISM devised using expression profile of 21 IFN-induced genes. In those with a high ISM at baseline, immunization with IFN-K significantly reduced this interferon gene signature. In these patients with a high ISM, treatment with IFN-K correlated significantly with greater decreases in ISM 16 weeks after starting treatment compared to those receiving placebo. No significant adverse events were observed other than injection site reactions of mild to moderate severity.

MEDI-546 is an antibody to type I IFN-α receptor that has been tried in patients with systemic sclerosis and has potential for investigation in patients with lupus.^31^ Another monoclonal antibody, AIA22, neutralizes multiple IFN-α subtypes and binds to a novel epitope on IFN-α which binds to the Type II IFN receptor.^32^ Its utility in lupus is being further investigated. Anifrolumab is another monoclonal antibody that targets interferon receptor Type I that is being currently investigated in lupus.^33^

## AGENTS CAUSING A DECREASE IN TYPE I INTERFERONS

Hydroxychloroquine (HCQ) is considered as one of the most important drugs in the management of lupus. Mechanistically, HCQ acts by interfering with endosomal interaction of nucleic acids (derived from apoptotic debris and immune complexes) with TLRs. This interrupts the loop driving type I interferon synthesis, which was responsible for creating a permissive environment for the production of autoantibodies. Indeed, treatment with HCQ has been shown to reduce levels of circulating Type I IFN.^15,34^ Bruton’s tyrosine kinase (Btk) is an enzyme involved in signal transduction in multiple immune cells. A recent paper^35^ demonstrated that inhibition of Btk by RN486 in purified human pDCs in-vitro resulted in abrogation of inflammatory signals downstream of TLR9 (including levels of IFN-α production), but not those following TLR7 stimulation. Blood dendritic cell antigen 2 (BDCA2) is an inhibitory receptor on human pDCs, which inhibits signaling via TLR7 and TLR9. A monoclonal antibody stimulating BDCA2^36^ was shown in-vitro in human pDCs derived from healthy controls as well as patients with lupus and in cynomolgus monkeys to inhibit production of Type I IFNs. This may be another potential method of reducing pathogenic Type I IFN secretion. Sphingosine-1 phosphate receptor (S1PR) is expressed on the surface of different immune cells including pDCs. Activation of S1PR ameliorates Type I IFN production resulting from stimulation of TLR7 or TLR9 in human pDCs,^37^ and serves as another potential target to reduce the secretion of Type I IFNs. Inhibitory oligonucleotides whose guanine moiety has been modified have been found to inhibit production of Type I IFNs by human pDCs as well as pDCs from MRL/lpr mice model of lupus which were stimulated via TLR7 and TLR9 agonists,^38^ identifying another potential therapeutic route in lupus.

Bortezomib is emerging as a promising agent for the treatment of refractory lupus, and acts by inhibiting the proteasome. A recent study^39^ of 12 patients with refractory lupus who received between one to four cycles of intravenous bortezomib (at a dose of 1.3 mg/m^2^) revealed not only a depletion of plasma cells (thought to be responsible for the beneficial effects of this drug in lupus) but also reduced the expression of Siglec-1 on monocytes, which is expressed following exposure to Type I IFNs.

Bcl-2 is an anti-apoptotic factor that plays a role in persistence of pathogenic pDCs, which are the source of Type I IFN in lupus. Studies in pDCs derived from NZB/W F1 mica as well as from humans (healthy controls and patients with lupus) reveal that exposure to Bcl-2 antagonists ABT-737 and ABT-199 reduces survival of these pDCs. This is another potential avenue to ameliorate the Type I IFN signature in lupus.^40^

Lipopolysaccharide (LPS) is a ligand for TLR4 and this further leads on to Type I IFN expression. Dihydroartemisinin, a commonly used anti-malarial drug, has been found to reduce secretion of IFN-α and IFN-β induced by LPS from splenocytes cultured from MRL/lpr mice.^41^ **Figure 1** summarizes the sites of action of these various therapies that have been discussed.

## CONCLUSION

Type I IFNs play a crucial role in driving the process of autoantibody generation in lupus. Patients with lupus have been found to exhibit an interferon signature metric that reflects activation of genes following stimulation by Type I IFNs. Although one would expect that interferon-targeted therapies should be more useful in those patients having a high interferon signature, the exact role of ISM in determining whether a patient will benefit with IFN-targeted therapies in unclear. For example, patients with a low ISM showed better response to rontalizumab^25^ than those with a high ISM. Hence, the use of ISM as a biomarker to decide the use of interferon-targeted therapies needs further exploration.

Monoclonal antibodies directly inhibiting Type I IFN (sifalimumab, rontalizumab, MEDI-546, AIA122), blocking of interferon receptor (anifrolumab) and stimulation of endogenous production of antibodies to Type I IFN by interferon alpha kinoid are promising strategies in the management of lupus and other autoimmune diseases. These drugs may be effective for inducing remission in difficult-to-treat lupus. There is inadequate literature to answer whether or not they may be useful for maintenance of remission. In addition, hydroxychloroquine and bortezomib also reduce the circulating levels of Type I IFN in lupus. Numerous other agents such as Bcl-2 antagonists, Btk inhibitors and dihydroartemisinin have shown promise in reducing levels of circulating Type I IFN in laboratory and animal models of lupus. IFN-blockade may be of value in those patients who are refractory to conventional induction regimens, however, their role in renal and neuropsychiatric lupus, which are two subtypes of lupus associated with higher morbidity, requires further exploration.

## ABBREVIATIONS:

APC: Antigen-presenting cell
BAFF: B-cell activating factor
BICLA: BILAG-based Composite Lupus Assessment
BILAG: British Isles Lupus Assessment Group
Btk: Bruton’s tyrosine kinase
CLASI: Cutaneous lupus erythematosus area and severity index
DNA: Deoxyribonucleic acid
EBV: Epstein Barr virus
EBNA1: EBV nuclear antigen 1
HCQ: Hydroxychloroquine
IFN: Interferon
IFN-K: Interferon α kinoid
ISG: Interferon-stimulated genes
ISM: Interferon signature metric
JIA: Juvenile idiopathic arthritis
LPS: Lipopolysaccharide
PBMC: Peripheral blood mononuclear cells
pDCs: plasmacytoid dendritic cells
pSLE: Pediatric SLE
RNA: Ribonucleic acid
S1PR: Sphingosine-1 phosphate receptor
SELENA-SLEDAI: Safety of Estrogens in Lupus Erythematosus National Assessment SLEDAI
SLE: Systemic lupus erythematosus
SLEDAI: SLE disease activity index
SLEDAI-2K: SLEDAI 2000
SRI: SLE responder index
USFDA: United States Food and Drug Administration
